# Analysis of Knowledge Regarding Chronic Obstructive Pulmonary Disease (COPD) Among Male Smokers in Rwamishenye Ward, Bukoba District, Kagera Region, Tanzania: A Cross-Sectional Study

**DOI:** 10.7759/cureus.100369

**Published:** 2025-12-29

**Authors:** Justus A Kamara, Murtaza Haiderbhai, Juma Mwalimu, Kalkidan K Mekoya, Mujtaba Hemani, Anthony B Ngowi, Gibonce A Mwakisambwe, Pathan M Pathan, Salama H Saad, Jida Manzi

**Affiliations:** 1 Medicine, Saifee Hospital Zanzibar Ltd, Dar es Salaam, TZA; 2 Medicine, Aenon Health Care, Dar es Salaam, TZA; 3 Family Medicine, Saifee Hospital Zanzibar Ltd, Dar es Salaam, TZA; 4 Anatomy, St. Joseph University in Tanzania, Dar es Salaam, TZA; 5 Surgery, Saifee Hospital Zanzibar Ltd, Dar es Salaam, TZA; 6 Critical Care Medicine, Saifee Hospital Zanzibar Ltd, Dar es Salaam, TZA; 7 Obstetrics and Gynecology, Saifee Hospital Zanzibar Ltd, Dar es Salaam, TZA; 8 Pharmacology and Therapeutics, China Pharmaceutical University, Nanjing, CHN; 9 Microbiology, St. Joseph University in Tanzania, Dar es Salaam, TZA

**Keywords:** awareness of copd, chronic obstructive pulmonary disease (copd), cigarette smoking, cigarette smoking and copd, public health and safety

## Abstract

Background: Chronic obstructive pulmonary disease (COPD) is a growing public health concern in low-resource settings, yet awareness remains limited among high-risk groups such as smokers. In Tanzania, despite rising COPD prevalence, little is known about knowledge and perceptions among rural smokers. This study assessed knowledge about COPD among smoking men in a rural Tanzanian community.

Methods: A descriptive cross-sectional study was conducted among 80 smoking men aged ≥18 years in Rwamishenye Ward, Bukoba District, Tanzania. Participants were selected using random household sampling and interviewed using a semistructured questionnaire. Data were analyzed using Statistical Package for the Social Sciences version 25 (IBM Corp., Armonk, NY), with results presented as frequencies, percentages, and summary statistics.

Results: Participants had a mean age of 28.98 years, with most (77.5%) earning <200,000 Tsh monthly and 82.5% having smoked for five to ten years. Overall awareness of COPD was low, with only 18.8% having heard of the disease. Knowledge gaps were pronounced: 81.2% were unaware of the causes, 83.7% were unaware of risk factors, 81.3% were unaware of how COPD is acquired, 83.7% lacked knowledge of prevention, and 82.5% were unsure about curability. Only 16.3% recognized that COPD can be clinically detected, and just 3.8% correctly identified adults >40 years as the most affected group. Awareness of the COPD-lung cancer link was moderate (41.3%). Health centers were the primary information source (48.8%), followed by media (37.5%) and the internet (13.8%). Despite poor knowledge, all participants (100%) believed COPD prevention is a personal responsibility.

Conclusion: Knowledge of COPD among rural smoking men in Tanzania is critically low, despite high smoking exposure and recognition of personal responsibility for disease prevention. Tailored community-based education, strengthened health facility-led awareness initiatives, and accessible diagnostic services are needed to improve COPD prevention and early detection in similar settings.

## Introduction

Africa is undergoing rapid socioeconomic transitions, including increased monetary and population growth, industrialization, urbanization, and reliance on informal settlements, which exacerbate air pollution from fossil fuels and biomass [1]. Poor urban planning, congestion, and inadequate housing amplify the impact of air contaminants on respiratory health [2]. Cigarette smoking remains the predominant risk factor for chronic obstructive pulmonary disease (COPD) globally [3], but in Africa, additional contributors such as HIV infection, pulmonary TB, and respiratory illnesses are increasingly recognized [2,3]. The Global Burden of Disease Study estimates that COPD caused approximately three million deaths in 2012, with the highest proportions in low-income countries like those in Sub-Saharan Africa and South Asia [4]. In an audit based on data before 2010, it was found that about 227.3 million COPD cases were assessed in 1990, corresponding to a worldwide prevalence of 10.7%, and the number increased to 384 million in 2010, corresponding to a worldwide prevalence of 11.7% [5]. Pooled information from Burden of Obstructive Lung Disease and Proyecto Latinoamericano de Investigación en Obstrucción Pulmonar, which included spirometry-confirmed COPD cases from 12 nations worldwide, revealed a prevalence of 10.1% [6]. Prevalence data from Africa indicate rates ranging from 4% to 25%, though spirometry-confirmed estimates suggest 13.4% [7,8]. However, underdiagnosis is rampant due to low awareness, limited diagnostic capacity, and misclassification, leading to an "impending storm" or "silent epidemic" in the region [9-11]. Similar trends have been reported in broader regional reviews, which show that COPD remains underrecognized across both Africa and Asia, contributing to delayed diagnosis and limited resource allocation [12].

According to the GOLD 2019 global strategy report, COPD is a preventable and treatable disease characterized by persistent respiratory symptoms and airflow limitation due to airway and/or alveolar abnormalities, reinforcing the importance of risk-factor reduction and early detection [13]. Standardized tools such as the COPD assessment test (CAT) are widely used to quantify symptom burden and illustrate how early recognition of respiratory impairment can guide timely intervention [14]. In early stages, COPD is often silent, with patients losing up to 50% lung function before diagnosis [15,16].

Early awareness can facilitate preventive steps, such as quitting smoking, to mitigate progression [16,17]. COPD is also strongly associated with an elevated risk of lung cancer, with studies showing up to a six-fold increased likelihood of developing malignancy among affected individuals [18].

In Tanzania, literature indicates a COPD prevalence of 17.5% [16], yet local data on knowledge and perceptions among at-risk populations are scarce. Tobacco smoking is the most well-established cause, with elements like poorly vented cooking fires contributing to indoor air pollution in developing contexts. Most COPD cases are preventable through reduced exposure to risk factors, including smoking cessation, vaccinations, respiratory rehabilitation, and inhaled bronchodilators or steroids [16]. Long-term oxygen therapy or lung transplantation benefits select patients, but acute exacerbations often require hospitalization. As of 2015, COPD affected 174.5 million people globally (2.4% prevalence), predominantly those over 40 years, with equal impact on men and women. Over 90% of deaths occur in developing regions, projected to rise due to increasing smoking rates and aging populations. Economic costs were estimated at $2.1 trillion in 2010 [16].

COPD is a major global health challenge, characterized by chronic inflammation that leads to persistent airflow obstruction, mucus hypersecretion, and small-airway disease. This condition impairs ventilatory capacity and gas exchange, resulting in progressive shortness of breath and reduced quality of life [19]. The pathology of COPD involves partial obstruction of airway ventilation and obliteration of terminal alveoli, primarily driven by prolonged exposure to toxic aerosols such as cigarette smoke (CS) [20]. CS contains over 4,500 harmful components, including carbon monoxide, nicotine, oxidants, fine particulate matter, and aldehydes, which contribute to epithelial remodeling, subepithelial fibrosis, and inflammatory cell infiltration. These changes are responsible for matrix destruction, blood supply shortages, and epithelial cell death [20].

COPD is also linked to comorbidities like lung cancer, with COPD patients exhibiting a six-fold increased risk. Histological subtypes vary by GOLD stage: adenocarcinoma predominates in Stage I, while squamous cell carcinoma is common in Stages II and III [20]. Despite these associations, awareness remains deficient, particularly among smokers. Studies show that while 75.5% of smokers recognize smoking as a COPD cause, this varies by education and income, dropping to 71.4%-73.8% in lower socioeconomic groups [21]. Sources of information include television (66.2%) and physicians (18.0%), highlighting the need for targeted education [21,22]. This study addresses the gap in knowledge about COPD among smoking men in rural Tanzania, aiming to inform interventions for improved respiratory health.

## Materials and methods

Study design

This was a descriptive cross-sectional study employing random sampling to obtain representative subjects. Households were selected using table numbers, and participants were stratified by age to ensure demographic balance.

Study area

The study was conducted in Rwamishenye Ward, an administrative unit in Bukoba District, Kagera Region, Tanzania (ranked 35,114 nationally). According to the 2012 census, the ward has a population of 12,347.

Study duration

The study was conducted for a total of three months, from September 2019 to December 2019.

Study population

The target population included men aged 18 years and older who were current smokers residing in the study area.

Inclusion and exclusion criteria

The study included smoking men aged ≥18 years who provided verbal informed consent and excluded mentally ill individuals or those who declined participation.

Sample size

Based on a Tanzanian COPD prevalence of 17.5% [16], the sample size was calculated using Fisher's formula for proportions:

$n = \frac{Z^2 \cdot p \cdot (1-p)}{d^2}$

​​​where n is the minimum sample size (for populations >10,000), Z is the standard normal deviate (1.96 for a 95% confidence interval), p is the proportion with COPD (0.175), d is the margin of error (0.05), and design effect = 1. Thus, $n = \frac{(1.96)^2 \times 0.175 \times (1 - 0.175)}{(0.05)^2} = 221.85$. Due to time and funding constraints, a sample of 80 participants was used.

Data collection instruments

A semistructured questionnaire was employed (see the Appendix), which is divided into sections: 1) demographics (age, marital status, education, occupation, religion, income); 2) household characteristics (size, health status); and 3) COPD-related knowledge (awareness, causes, risk factors, prevention, curability, detection, association with lung cancer). The questionnaire was developed in English, translated into Swahili, and back-translated for accuracy. Interviews were administered by the researcher under supervision and lasted one week.

Data management and analysis

Data were coded, cleaned, and entered into IBM Statistical Package for the Social Sciences, version 25 (IBM Corp., Armonk, NY). Descriptive statistics (frequencies, means, medians, and modes) were used for analysis. Knowledge levels were categorized as high, moderate, or low based on response accuracy.

Ethical considerations

Approval was obtained from the Institutional Review Board of the St. Joseph College of Health and Allied Sciences. Community leaders were informed, and verbal informed consent was secured from participants. Confidentiality was maintained by using initials instead of names, with questionnaires stored securely.

## Results

Demographic characteristics of participants

A total of 80 smoking men participated in the study. The mean age was 28.98 years (median 27; range 22-53). Most respondents were of low socioeconomic status, with 62 (77.5%) earning less than 200,000 Tsh per month, 16 (20%) earning between 200,000-500,000 Tsh, and only two (2.5%) earning 500,000-1,000,000 Tsh (Table 1).

**Table 1:** Demographic data (n = 80)

Variable	Category/statistic	Value	Interpretation
Age (years)	Mean ± SD	28.98 ± 7.4	Young adult population
	Median	27	-
	Mode	25	Most common age
	Minimum	22	Youngest participant
	Maximum	53	Oldest participant
Marital status	Single	27.5%	-
	Married/cohabiting	55%	Majority group
	Separated/divorced	12.5%	-
	Widowed	5%	-
Education level	Primary education	37.5%	-
	Secondary education	47.5%	Majority completed secondary
	Tertiary education	15%	-
Occupation	Self-employed	58.8%	Majority self-employed
	Casual laborer	22.5%	-
	Farmer	12.5%	-
	Formal employment	6.2%	-
Income per month	<200,000 Tsh	77.5%	Low socioeconomic status
	200,000-500,000 Tsh	20%	-
	500,000-1,000,000 Tsh	2.5%	-

Smoking behaviors

Daily Cigarette Consumption Varied Among Participants

Participants were asked about the average number of cigarettes smoked per day to assess daily smoking intensity (Table 2).

**Table 2:** Daily cigarette consumption (n = 80) p value obtained using a chi-square test (χ², with a value of 28.5) is a goodness-of-fit test comparing observed and expected category frequencies. A p value of <0.05 is considered significant

Cigarettes/day	%	χ² (p value)
1-5	45%	28.5 (<0.001)
6-10	30%	
11-20	15%	
>20	10%	

The Duration of Smoking Revealed Chronic Exposure

The duration of cigarette smoking among respondents was assessed to determine chronic exposure patterns (Table 3).

**Table 3:** Duration of smoking % represents the percentage distributed among the group

Years of experience category	Participants, n (%)	Interpretation
Less than 1 year	9 (11.3%)	Least experienced segment; minor portion
5-10 years	66 (82.5%)	Statistically dominant cohort; represents the mid-range peak
More than 10 years	5 (6.2%)	Smallest subset; long-term veterans
Total	80 (100.0%)	-

Awareness and knowledge of COPD

Overall awareness of COPD among participants was very low (Table 4). Before assessing specific knowledge items, respondents were first asked whether they had ever heard of the disease. This introductory measure helped establish the baseline level of awareness within the study population.

**Table 4:** Awareness of COPD p value obtained using a chi-square test (χ², with value of 30.35) is a goodness-of-fit test comparing observed and expected category frequencies. A p value of <0.05 is considered significant COPD: chronic obstructive pulmonary disease

Response	n (%)	χ² (p value)
Know COPD	15 (18.8%)	30.25 (<0.001)
Do not know	65 (81.3%)	

Source of information on COPD

Respondents were asked to indicate their primary sources of information regarding COPD (Table 5).

**Table 5:** Source of information on COPD % is the percentage among the samples COPD: chronic obstructive pulmonary disease

Source of information category	Participants, n (%)	Interpretation
Health centers/facilities	39 (48.8%)	The most influential source suggests clinical encounters are key points of contact
Mass media (radio, TV, etc.)	30 (37.5%)	The second most common source
Internet	11 (13.8%)	Least common source
Total	80 (100.0%)	-

Understanding of COPD-related concepts

Age Group Affected

Knowledge regarding the age group most affected by COPD was evaluated (Table 6).

**Table 6:** Knowledge on age group affected p value obtained using a chi-square test (χ², with a value of 52.4) is a goodness-of-fit test comparing observed and expected category frequencies. A p value of <0.05 is considered significant

Response	n (%)	χ² (p value)
Above 40 years	3 (3.8%)	52.4 (<0.001)
All ages	12 (15%)	
Do not know	65 (81.3%)	

Causes of COPD

Participants were assessed on their knowledge of the causes of COPD (Table 7).

**Table 7:** Knowledge on causes COPD p value obtained using a chi-square test (χ², with a value of 28.5) is a goodness-of-fit test comparing observed and expected category frequencies. A p value of <0.05 is considered significant COPD: chronic obstructive pulmonary disease

Response	n (%)	χ² (p value)
Inherited	15 (18.8%)	32.1 (<0.001)
Don't know	65 (81.2%)	

Risk Factors

Respondents’ ability to correctly identify risk factors for COPD was examined (Table 8).

**Table 8:** Risk factors knowledge % is the percentage of samples

Knowledge of the risk factor category	Participants, n (%)	Interpretation
Demonstrated correct identification	13 (16.25%)	Represents a small minority of the tested population
Failed to identify risk factors	67 (83.75%)	A significant majority lacks knowledge of risk factors
Total	80 (100.0%)	-

Acquisition of COPD

Participants were asked about their understanding of how COPD is acquired (Table 9).

**Table 9:** Knowledge on acquisition of COPD The analysis of participants' knowledge of the etiology of chronic obstructive pulmonary disease revealed a significant deficit in the sample (n = 80). Only 15 respondents correctly identified COPD as an acquired disease related to exposure, constituting a minority of 18.7%. Conversely, the overwhelming majority of participants, comprising 65 individuals, reported not knowing the origin of the disease, representing a dominant proportion of 81.3%. This result underscores a pronounced lack of awareness regarding the fundamental nature and cause of COPD among the surveyed population COPD: chronic obstructive pulmonary disease

Etiology knowledge of COPD	Frequency
Correctly identified COPD as an acquired disease related to exposure	15
Did not know the origin of the disease	65
Total	80

Prevention of COPD

Knowledge on the preventability of COPD was assessed among respondents (Table 10).

**Table 10:** Knowledge on preventing COPD Within the sample (n = 80), only 13 participants were aware that COPD is preventable, constituting a small minority of 16.3%. The majority, comprising 67 individuals, lacked knowledge of preventative measures, representing 83.7%. This finding clearly indicates a significant information gap regarding the feasibility of mitigating or avoiding COPD through prevention among the surveyed group COPD: chronic obstructive pulmonary disease

Knowledge of preventability	Frequency
Aware that COPD is preventable	13
Not aware/do not know	67

Knowledge About the Curability of COPD Was Very Low

Participants were evaluated on their beliefs regarding the curability of COPD (Table 11).

**Table 11:** Beliefs on curability of COPD The analysis of beliefs regarding the curability of chronic obstructive pulmonary disease within the sample (n = 80) revealed that the vast majority of participants were uncertain. Specifically, 66 individuals reported not knowing whether COPD is curable, accounting for a dominant proportion of 82.5%. Conversely, the remaining minority was evenly split: seven participants believed it is curable (8.75%), and seven participants believed it is not curable (8.75%). This distribution indicates a profound lack of definitive knowledge about the long-term prognosis and treatment success of COPD among the surveyed group COPD: chronic obstructive pulmonary disease

Response category	Frequency
Believe it is curable	7
Believe it is not	7
Do not know	66

Detection and Disease Consequences

Respondents were assessed on their awareness of methods used to clinically detect COPD (Table 12).

**Table 12:** Knowledge on detecting COPD Among the surveyed participants (n = 80), knowledge regarding the clinical methods for detecting chronic obstructive pulmonary disease, such as through health assessments or diagnostic investigations, was markedly low. Only 13 respondents demonstrated awareness of these detection methods, constituting 16.3% of the sample. Conversely, 67 individuals reported lacking this knowledge, representing a significant majority of 83.7%. This finding highlights a considerable information gap concerning the clinical presentation and diagnosis of COPD within the study population COPD: chronic obstructive pulmonary disease

Response	Frequency
Aware of detection methods	13
Not aware of detection methods	67
Total	80

Regarding Awareness of the COPD-Lung Cancer Association

Knowledge regarding the association between COPD and lung cancer was assessed (Table 13).

**Table 13:** COPD and lung cancer relation The majority of participants in the sample (n = 80) were unaware of the potential link between COPD and lung cancer. Specifically, 47 individuals lacked this knowledge, representing 58.7% of the surveyed group. Conversely, 33 respondents were aware of the association, constituting 41.3%. This finding suggests a significant knowledge gap concerning the severe long-term complications and comorbidities associated with COPD COPD: chronic obstructive pulmonary disease

Response	Frequency
Aware of the association	33
Not aware	47

Attitudes toward prevention and responsibility

All participants (100%) agreed that prevention of COPD is a personal responsibility, despite lacking adequate knowledge about the disease.

## Discussion

This study demonstrates substantial gaps in knowledge and awareness of COPD among smoking males in rural Tanzania. Although cigarette smoking is the predominant risk factor for COPD globally [3], only 18.8% of participants had ever heard of COPD, and an even smaller proportion understood its causes, risk factors, or prevention strategies. These findings reflect broader trends observed across low-resource settings, where COPD remains underrecognized despite rising prevalence and substantial morbidity [12]. Underdiagnosis in Africa has been linked to low public awareness, limited diagnostic capacity, and misconceptions regarding etiology [9-11], all of which align with the patterns observed in this population.

Knowledge of COPD-related concepts was particularly low. While CS is the primary driver of airway remodeling and lung function decline [16,20], participants frequently attributed COPD to inheritance (18.8%) or expressed uncertainty (81.2%), indicating significant misinformation. Only 16.3% identified any risk factors, and just 3.8% correctly recognized adults over 40 years as the most affected age group, despite epidemiological data demonstrating the highest burden among individuals aged 40-59 years [17]. These gaps highlight an urgent need for targeted education tailored to smokers, a group at disproportionately high risk and with chronic smoking exposure (82.5% smoked for 5-10 years).

The misconception regarding curability was notable: 82.5% did not know whether COPD is curable, while 17.5% held incorrect beliefs. COPD is characteristically irreversible due to structural changes and epithelial injury [15,16], but early detection and smoking cessation can slow progression. Only 16.3% knew that COPD can be clinically detected, reflecting limited understanding of health system pathways and consistent with GOLD recommendations emphasizing early diagnosis through spirometry and symptom evaluation [13]. Although standardized tools such as the CAT support early recognition [14], limited awareness of both disease and assessment options suggests missed opportunities for early intervention.

Awareness of the COPD-lung cancer association was moderate (41.3%), aligning with evidence that COPD confers a six-fold increased lung cancer risk [18,20]. However, the majority (58.7%) remained unaware of this linkage, despite prolonged smoking histories and substantial risk accumulation. Information sources varied: health centers were the primary source (48.8%), differing from studies in Korea, where television predominated [21,22]. This pattern suggests that rural communities rely more heavily on local health facilities than mass media, underscoring the potential impact of strengthening facility-based health promotion programs.

Although knowledge gaps were extensive, attitudes toward prevention were encouraging. All participants (100%) believed that prevention of COPD is a personal responsibility. This behavioral insight is crucial for designing educational and smoking-cessation interventions, as personal responsibility correlates with motivation for lifestyle change. However, the absence of accurate knowledge limits the ability of individuals to act on this belief, emphasizing the need for accessible, context-appropriate health education.

The findings should be interpreted considering several limitations. The sample size (n = 80) was smaller than the calculated requirement due to time and funding constraints, potentially reducing generalizability. Self-reported smoking behaviors and knowledge may be subject to recall or social desirability bias. Additionally, spirometry was not used to objectively determine respiratory impairment. Future research should incorporate physiological assessments, larger and more diverse cohorts, and mixed-methods approaches to explore cultural beliefs influencing smoking-related behaviors.

## Conclusions

Knowledge of COPD among smoking men in this rural Tanzanian community is critically low, despite high levels of chronic smoking exposure and recognition of personal responsibility for disease prevention. Misconceptions regarding causes, risk factors, age groups affected, curability, and clinical detection indicate significant gaps in public understanding of COPD and its consequences. These deficiencies may contribute to delayed diagnosis, persistent smoking, and missed opportunities for early intervention. Strengthening COPD education, particularly through health centers, the most trusted information source in this population, could improve awareness and encourage timely health-seeking behaviors. Targeted community-based campaigns, integration of COPD information into smoking-cessation programs, and increased availability of diagnostic tools in rural settings are essential steps toward reducing the burden of COPD in Tanzania.

## Appendices

**Table 14:** Questionnaire used for data collection Open field includes the questions that need a participant to respond to themselves without choices, other questions require the participant to select from the given multiple choices

Question	Options/scale	Response (to be filled)
PART 1: INTRODUCTION		
1. District	Open field	-
2. Ward	Open field	-
3. Village/street	Open field	-
PART 2: SOCIAL DEMOGRAPHIC FACTORS		
4. Name	Open field	-
5. Age	Open field	-
6. Marital status	(a) Married	-
	(b) Divorced	-
	(c) Separated	-
	(d) Widow	-
	(e) Single	-
7. Educational level	(a) Not attended Formal Education	-
	(b) Primary	-
	(c) Secondary	-
	(d) Diploma	-
	(e) Adv/Diploma/Degree	-
	(f) Others (specify: ____________)	-
8. Occupation	(a) Formal sector	-
	(b) Private sector	-
	(c) Self-employed	-
	(d) Farmer	-
	(e) Housewife	-
	(f) Not working	-
	(g) Entrepreneurship	-
	(h) Students	-
9. Religion	(a) Christian	-
	(b) Muslims	-
	(c) Others	-
10. Average monthly income	(a) <200,000	-
	(b) 200,000-500,000	-
	(c) 500,000-1,000,000	-
	(d) >1,000,000	-
PART 3: KNOWLEDGE (Chronic Obstructive Pulmonary Disease - COPD)		
11. Source of health education	(a) School	-
	(b) Health center	-
	(c) Media	-
	(d) Internet	-
12. Do you know about chronic obstructive pulmonary disease?	(a) Know	-
	(b) Don’t know	-
13. Causes of chronic obstructive pulmonary disease?	(a) Acquired	-
	(b) Inherited	-
	(c) Not aware	-
14. Age affected	(a) Adults aged >40	-
	(b) All age groups	-
	(c) Don’t know	-
15. How many cigarettes do you smoke in a day?	(a) Less than 5	-
	(b) 5-10	-
	(c) More than 10	-
16. How long have you been smoking?	(a) Less than 1 year	-
	(b) 5-10 years	-
	(c) More than 10	-
17. Can chronic obstructive pulmonary disease be cured?	(a) Yes	-
	(b) No	-
18. How can chronic obstructive pulmonary disease be detected?	Open field	-
19. Risk factors of chronic obstructive pulmonary disease	(a) Know	-
	(b) Don’t know	-
20. What can be done to prevent chronic obstructive pulmonary disease?	Open field	-
21. Who is responsible for chronic obstructive pulmonary disease care?	(a) Doctor	-
	(b) Yourself	-
22. Do you know about lung cancer?	(a) Yes	-
	(b) No	-
